# Development and characterization of GR2E Golden rice introgression lines

**DOI:** 10.1038/s41598-021-82001-0

**Published:** 2021-01-28

**Authors:** B. P. Mallikarjuna Swamy, Severino Marundan, Mercy Samia, Reynante L. Ordonio, Democrito B. Rebong, Ronalyn Miranda, Anielyn Alibuyog, Anna Theresa Rebong, Ma. Angela Tabil, Roel R. Suralta, Antonio A. Alfonso, Partha Sarathi Biswas, Md. Abdul Kader, Russell F. Reinke, Raul Boncodin, Donald J. MacKenzie

**Affiliations:** 1 International Rice Research Institute (IRRI), DAPO Box 7777, Metro Manila, Philippines; 2 Philippines Rice Research Institute (PhilRice), Maligaya, Science City of Munoz, Philippines; 3 Plant Breeding Division, Bangladesh Rice Research Institute (BRRI), Gazipur, Bangladesh; 4 Institute for International Crop Improvement, Donald Danforth Plant Science Center, Saint Louis, MO USA

**Keywords:** Biotechnology, Genetics, Plant sciences

## Abstract

Golden Rice with β-carotene in the grain helps to address the problem of vitamin A deficiency. Prior to commercialize Golden Rice, several performance and regulatory checkpoints must be achieved. We report results of marker assisted backcross breeding of the GR2E trait into three popular rice varieties followed by a series of confined field tests of event GR2E introgression lines to assess their agronomic performance and carotenoid expression. Results from confined tests in the Philippines and Bangladesh have shown that GR2E introgression lines matched the performance of the recurrent parents for agronomic and yield performance, and the key components of grain quality. Moreover, no differences were observed in terms of pest and disease reaction. The best performing lines identified in each genetic background had significant amounts of carotenoids in the milled grains. These lines can supply 30–50% of the estimated average requirements of vitamin A.

## Introduction

Rice (*Oryza sativa*) is the major source of energy and nutrition for more than half the world’s population^1^. However, rice supplies minimal micronutrients in its milled form and completely lacks β-carotene which is the precursor for vitamin A. Thus, resource-poor people primarily dependent on rice with little access to diverse diets suffer from micronutrient deficiencies, also termed hidden hunger^2,3^. Even though efforts are being made to address micronutrient deficiencies by supplementation, fortification, and dietary diversification, the problem still persists globally. Biofortification of major staple crops has been recognized as one of the sustainable means to tackle micronutrient deficiencies especially in the vulnerable target groups in rural areas^4^.

Vitamin A is essential for various functions in the human body such as development and functioning of the visual system, differentiation and maintenance of cells, epithelial membrane integrity, and production of red blood cells, immune system, reproduction, and iron metabolism^5,6^. An estimated 190 million children and 19 million pregnant women have vitamin A deficiency (VAD), and almost a million children go blind every year^7^. In the Philippines, VAD ranges between 19.6 to 27.9% in infants and preschool children^8^, while in Bangladesh, over half of the preschool age (56.3%) and school age children (53.3%) at the national level were found to exhibit at least a mild grade of VAD^9^.

Several crops such as maize, cassava, and sweet potato have been successfully biofortified with elevated levels of provitamin A^10,11^. However, there is no naturally-occurring variation for provitamin A in grains in rice germplasm, so this has been achieved by using genetic engineering approaches. The genetic modification was made by the addition of two genes, phytoene synthase (*Zmpsy1*) from *Zea mays* and carotene desaturase (*crtI*) gene from the common soil bacterium, *Pantoea ananatis* (syn. *Erwinia uredovora*) into a temperate *japonica* rice variety, Kaybonnet, from the USA. This completed the carotenoid pathway in the grain and resulted in the accumulation of β-carotene in the endosperm^12,13^. However, the transfer of this golden rice trait from Kaybonnet into additional locally-adapted and widely-grown rice varieties is required for the successful release and adoption of golden rice in Asia.

Among the six second-generation Golden Rice (GR2) events received by the International Rice Research Institute (IRRI), event GR2E was found to contain a single intact copy of the inserted DNA integrated at a single site within the rice genome, giving rise to agronomically desirable progeny with suitable grain carotenoid content. This event has been transferred into Asian rice varieties through marker-assisted backcrossing (MABC). MABC has been successfully used to transfer high value genes/QTLs for disease resistance, submergence and drought tolerance traits into popular rice varieties without altering their desirable traits^14–16^.

Development of stable golden rice breeding lines with nutritionally relevant levels of provitamin A and without trait-associated yield, grain quality, or disease resistance penalties relative to the recipient parental varieties is essential for the successful adoption of golden rice. Introgression of the GR2E locus from GR2E Kaybonnet into PSBRc82, IR64, and BRRI dhan29 (BR29) was performed at IRRI through MABC along with selections for desirable agronomic and grain quality traits. The phenotypic evaluation was conducted under screen house conditions. Selection of homozygous plants and lines were carried out under field conditions at IRRI. Agronomic evaluations of selected lines were carried out under field conditions in a series of confined field tests (CTs) at IRRI, PhilRice and BRRI.

The main objectives of the present work were to: develop agronomically desirable lines of provitamin A enriched GR2E golden rice in the genetic backgrounds of popular rice varieties from Asia; to understand the effects of genetic background and environment on carotenoid expression, and to identify stable and productive lines of GR2E golden rice for varietal evaluation.

## Results

### Introgression of event GR2E into multiple genetic backgrounds

**Figure 1:**
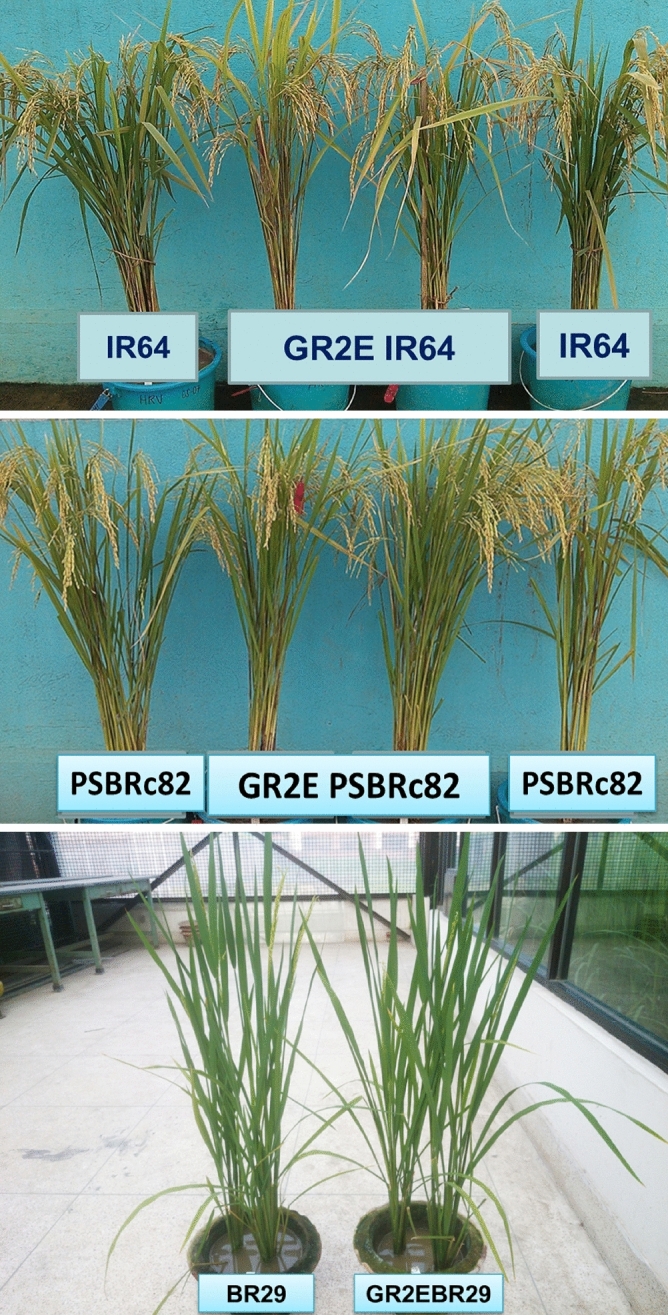
(**a**–**c**) GR2E introgression lines.

A series of five backcrosses of event GR2E Kaybonnet into three widely-grown rice varieties, IR64, PSBRc82, and BR29, resulted in the identification of introgression lines that were agronomically similar to their respective recipient parents. The stability and inheritance of the GR2E locus was confirmed using event-specific PCR in every generation, where it was found to segregate without distortion in a typical 1:1 Mendelian ratio in all the backcross generations (BC_1_ to BC_5_) and genetic backgrounds. All seeds containing the GR2E event showed the typical golden yellow color, indicating the expression of the provitamin A trait in the endosperm. Hemizygous (It is a condition in a diploid organism, where only one copy of the locus is present) plants phenotypically similar to their respective recipient parents were identified, backcrossed and advanced up to BC_5_F_1_, and with each successive backcross there was a progressive increase in similarity of the progenies to their respective recurrent (recipient) parents (Fig. 1). A total of 400, 190, and 94 BC_5_F_1_ plants of IR64, PSBRc82, and BR29, respectively, were phenotyped and genotyped by event-specific PCR. Yellow BC_5_F_2_ seeds were selected and analyzed for total carotenoid content, which ranged from 3.6–6.2 ppm in IR64, 3.1–6.4 ppm in PSBRc82, and 3.2–8.0 ppm in BR29. The BC_5_F_2_ plants were closer to respective recipient parents for key agronomic traits with average days to flowering (DTF), plant height (PH) and number of panicles (NP) of the selected BC_5_ progenies were 71.5 days, 108 cm and 15 for IR64, 82.5 days, 122.3 cm and 15.4 for PSBRc82 and 83 days, 117 cm and 17 for BR29 respectively. The final set of BC_5_F_3_ selected lines had background recovery of more than 98%. Agro-morphological traits, panicle characteristics, and grain parameters were similar to the recipient parents and no unintended, unexpected, effects due to the presence of the GR2E event were observed throughout the backcross breeding program. Based on the overall agronomic performance, carotenoid levels, and genetic background recovery, 40 BC_5_F_1_ plants in the IR64 background, and 20 BC_5_F_1_ plants in each of the PSBRc82 and BR29 backgrounds were selected. The BC_5_F_2_ seeds produced by each of these plants were further evaluated under field conditions in confined tests and plants homozygous for the GR2E locus were selected.

### Selection of homozygous and agronomically acceptable GR2E lines

The first confined field test of GR2E breeding lines was carried out during the 2015WS at IRRI to make individual homozygous plant selections. From among 8000 BC_5_F_2_ plants tested, a total of 602, 439, and 471 plants homozygous for the GR2E locus were identified in IR64, PSBRc82, and BR29, respectively (Fig S1). Efforts were focused on the lines homozygous for GR2E; however, hemizygous and null plants were also phenotyped to determine the impact of the presence of the GR2E locus on agronomic traits. The pair-wise t-tests were conducted between families derived from single BC_5_F_1_ plants within each of the three genetic backgrounds. Significant differences between families for total carotenoids were noted in a number of the possible pair-wise comparisons (data not shown). The mean comparisons between homozygous, hemizygous and null GR2E plants within each of the three populations did not show any abnormal deviations for key agronomic traits (Fig S2). The mean PH of lines carrying GR2E were marginally shorter than the respective recipient parent. For the remaining traits there were no clear differences between plants carrying GR2E and the respective parent variety. A total of 70 BC_5_F_3_ ILs similar to their respective parents and having higher levels of carotenoids were selected for IR64 and PSBRc82 genetic backgrounds.

### Evaluation of GR2E introgression lines in multi-location replicated confined tests

**Figure 2:**
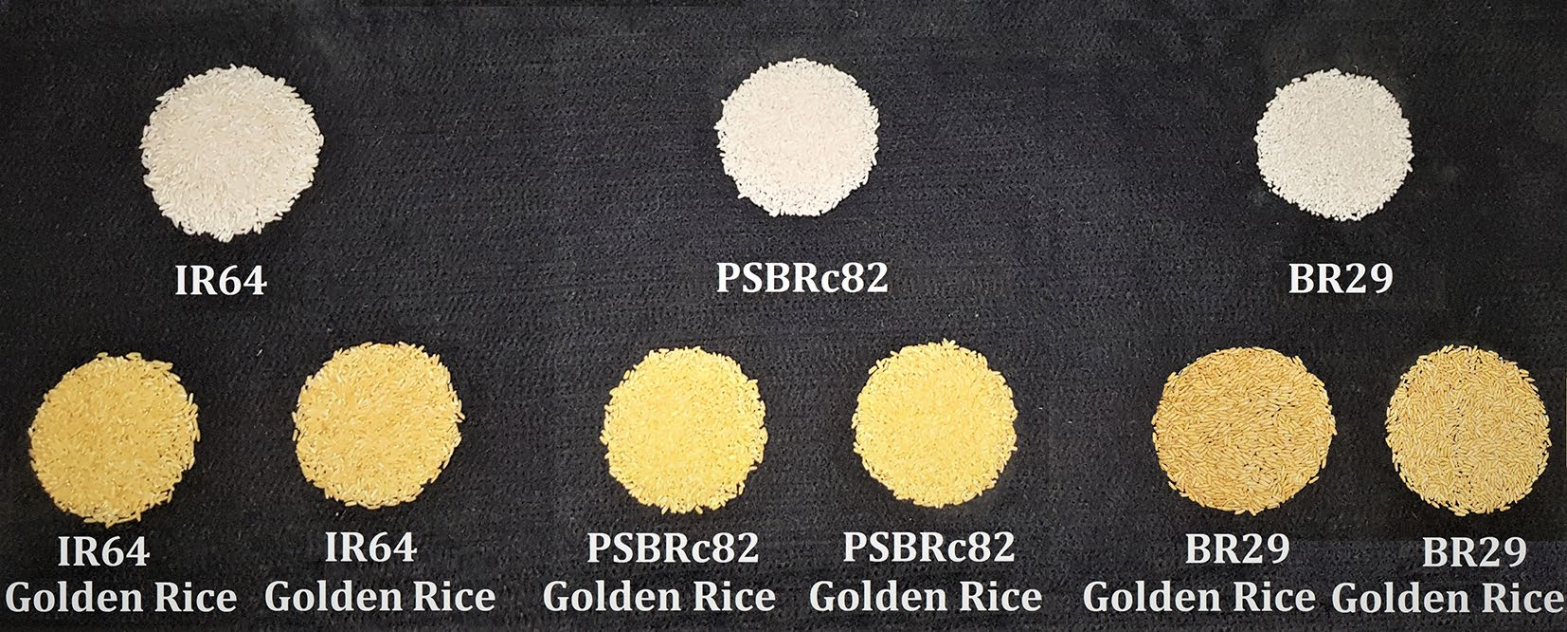
Grain samples of GR2E golden rice and respective recipient parents.

Agronomic performance of GR2E Introgression Lines (ILs) and their respective control varieties were assessed in a series of CTs at IRRI (2015WS, 2016DS and 2016WS), PhilRice (2015WS and 2016DS) and BRRI in Bangladesh (2016 Boro). A total of 70 ILs similar to their respective parents in agronomic performance and having the greatest levels of carotenoids were selected from each of IR64 and PSBRc82 backgrounds. A total of 14 agronomic, yield and yield-related traits and carotenoid content were measured from the different confined tests. Among the 70 ILs tested during the 2015WS at IRRI, PSBRc82 GR2E ILs showed small but statistically significant differences from non-transgenic PSBRc82 for eight traits including days to flowering (DTF), plant height (PH), Flag leaf length (FL), flag leaf width (FW), filled spikelets (FS), total number of spikelets per plant (TSP), grain length (GL) and hundred seed weight (HSW) (Table 1). However, in successive CTs conducted using 32 GR2E PSBRc82 ILs at IRRI and PhilRice, only FL, GL and HSW (2016DS), and GL, HSW and plot yield (PY) (2016WS; IRRI) showed significant differences. On the other hand, no significant differences were observed during the 2016DS and only GL and HSW showed significant differences at PhilRice in 2016WS (Table 2). Similarly GR2E IR64 ILs showed small but significant differences to the recipient parent for FL, TSP, GL, GW and HSW in 2015DS and for FW, FS, spikelet fertility (SF) and PY in 2016DS, while only GL showed significant difference in 2016WS. For the CT conducted with GR2E BR29 ILs in Bangladesh in the 2016 Boro season there were no significant differences from BR29 for all the traits measured (Table 3). Significant variations in total carotenoids among different families were observed in all backgrounds. The highest concentration of total carotenoids was observed in the BR29 background, followed by the PSBRc82 background, while the IR64 background had the lowest concentration of total caroteneoids (Tables 1, 2, 3). The grain samples of GR2E ILs along with recipient parents are shown in Fig. 2. Grain quality traits amylose content (AC), gel consistency (GC) and alkali spreading value (ASV) were measured for PSBRc82, IR64 and BR29 (Tables 1, 2, 3). There were no significant differences for AC between GR2E PSBRc82 ILs and PSBRc82 in all the trials. There were no significant differences in ASV and AC between GR2E IR64 ILs and the IR64 parent, while for BR29 there were no differences between the transgenic and the control except for AC. The background recovery of final set of selected BC_5_F_3_ ILs showed more than 98% recipient genome in all the three genetic backgrounds (Fig S3–S5). There was no significant difference in AC except in BR29, similarly for GC some minor significant differences were observed in PSBRc82 and IR64 in some seasons.

**Table 1:** Agronomic and grain quality performance of GR2EPSBRc82 at IRRI.

Traits	2015WS-IRRI			2016DS-IRRI			2016WS-IRRI		
	GR2EPSBRc82	PSBRc82	p value	GR2EPSBRc82	PSBRc82	p value	GR2EPSBRc82	PSBRc82	p value
DTF	83.2 ± 0.1	86.7 ± 0.3	0.000	79.2 ± 0.1	78.3 ± 0.8	0.325	89.4 ± 0.3	87.0 ± 1.7	0.179
PH (cm)	126.2 ± 0.4	131.1 ± 1.0	0.012	95.5 ± 0.4	98.5 ± 2.0	0.145	132.0 ± 0.5	136.2 ± 2.7	0.128
TN	13.7 ± 0.2	13.1 ± 0.5	0.952	15.1 ± 0.2	17.2 ± 1.1	0.068	14.0 ± 0.2	13.2 ± 1.1	0.474
PN	13.0 ± 0.2	12.2 ± 04	0.809	13.8 ± 0.2	15. 7 ± 1.1	0.065	13.6 ± 0.2	12.9 ± 1.1	0.512
PL (cm)	25.5 ± 0.1	25.5 ± 0.2	0.663	23.8 ± 0.1	23.5 ± 0.6	0.699	26. 5 ± 0.2	27.7 ± 1.1	0.299
FL (cm)	36.5 ± 0.4	32.5 ± 0.9	0.024	40.4 ± 0.5	33.7 ± 2.7	0.015	42.0 ± 0.5	38.6 ± 3.0	0.282
FW(cm)	1.6 ± 0.01	1.5 ± 0.02	0.028	1.3 ± 0.03	1.6 ± 0.2	0.245	1.5 ± 0.008	1.5 ± 0.05	0.283
FS	1298.6 ± 19.3	1099.9 ± 44.9	0.029	1538.5 ± 22.5	1477.9 ± 127.0	0.640	1176. 1 ± 21.4	1155.8 ± 120.9	0.869
TSP	1806.3 ± 24.7	1485.0 ± 57.7	0.002	1892.7 ± 29.0	1852.1 ± 164.1	0.808	1725.1 ± 25.5	1501.4 ± 144.1	0.130
SF (%)	72.0 ± 0.6	74.1 ± 1.5	0.131	81.5 ± 0.5	80.4 ± 2.7	0.677	68.5 ± 1.0	77.1 ± 5.6	0.135
GL(mm)	9.2 ± 0.02	9.8 ± 0.06	0.000	9.0 ± 0.03	9.6 ± 0.2	0.001	9.2 ± 0.03	9.7 ± 0.2	0.003
GW (mm)	2.5 ± 0.005	2.5 ± 0.01	0.179	2.4 ± 0.005	2.4 ± 0.02	0.353	2.5 ± 0.008	2.4 ± 0.04	0.042
HSW (g)	2.6 ± 0.01	2.5 ± 0.02	0.000	2.5 ± 0.01	2.6 ± 0.07	0.014	2.5 ± 0.02	2.8 ± 0.09	0.012
PY (g)	337.9 ± 4.9	327.5 ± 11.4	0.121	807.5 ± 6.3	821.0 ± 35.8	0.711	705.6 ± 12.0	878.1 ± 68.0	0.014
TC (ppm)	4.4 ± 0.06	–	0.000	4.7 ± 0.06	–	0.000	5.0 ± 0.1	–	0.000
AC (%)	20.6 ± 0.2	19.2 ± 1.1	0.248	22.8 ± 0.2	22.9 ± 0.9	0.909	23.1 ± 0.3	22.7 ± 1.6	0.666
GC	59.7 ± 1.6	62.7 ± 9.1	0.750	82.4 ± 1.5	84.3 ± 8.7	0.830	68.4 ± 1.6	77.7 ± 9.2	0.325
ASV	HI/I	HI/I	–	HI/I	HI/I	–	HI/I	HI/I	–

*DTF* days to fifty percent flowering, *PH* plant height, *TN* Tiller number, *PN* Panicle number, *PL* Panicle length, *FL* flag leaf length, *FW* flag leaf width, *FS* number of filled spikelets, *TSP* total number of spikelets per plant, *SF* spikelet fertility, *GL* grain length, *GW* grain width, *HSW* hundred seed weight, *PY* plot yield, *TC* total carotenoids, *AC* amylose content, *GC* gel consistency, *ASV* alkali spreading value.

**Table 2:** Agronomic and grain quality performance of GR2EPSBRc82 at PhilRice.

Traits	2016DS-PhilRice			2016WS-PhilRice		
	GR2E PSBRc82	PSBRc82	p value	GR2E PSBRc82	PSBRc82	p value
DTF	86.0 ± 0.2	85.8 ± 0.6	0.231	81.5 ± 0.3	81.3 ± 1.4	0.985
PH (cm)	130.5 ± 0.4	129.2 ± 1.5	0.276	96.1 ± 0.4	98.3 ± 2.1	0.288
TN	9.9 ± 0.1	9.4 ± 0.4	0.357	10.8 ± 0.1	10.7 ± 0.8	0.867
PN	9.7 ± 0.1	9.4 ± 0.4	0.361	10.8 ± 0.1	10.7 ± 0.8	0.875
PL (cm)	26.7 ± 0.1	26.6 ± 0.5	0.093	24.1 ± 0.2	23.7 ± 1.4	0.792
FL (cm)	31.9 ± 0.4	31.6 ± 1.2	0.687	27.9 ± 0.3	25.8 ± 1.7	0.236
FW(cm)	1.3 ± 0.01	1.3 ± 0.03	0.643	1.2 ± 0.007	1.1 ± 0.05	0.205
FS	1098. 2 ± 20.1	1145.8 ± 69.8	0.781	1274.6 ± 27.8	1007.0 ± 158.0	0.085
TSP	1262. 9 ± 21.3	1329.0 ± 74.0	0.754	1410.1 ± 30.7	1089.2 ± 174.5	0.063
SF (%)	86.9 ± 0.5	86.3 ± 1.8	0.060	90.4 ± 0.4	92.6 ± 2.3	0.376
GL(mm)	9.7 ± 0.4	9.3 ± 1.5	0.149	8.9 ± 0.02	9.5 ± 0.1	*0.000*
GW (mm)	2.4 ± 0.01	2.5 ± 0.03	0.233	2.6 ± 0.01	2.5 ± 0.08	0.555
HSW (g)	2.6 ± 0.1	2.5 ± 0.4	0.951	2.4 ± 0.01	2.6 ± 0.07	*0.002*
PY (g)	376. 8 ± 5.0	404. 9 ± 17.4	0.583	654.7 ± 9.8	681.3 ± 55.5	0.658
TC (ppm)	4.8 ± 0.06	–	0.000	4.1 ± 0.05	–	0.000
AC (%)	20.0 ± 0.2	19.3 ± 1.4	0.644	20.6 ± 0.2	19.2 ± 1.1	0.248
GC	65.3 ± 1.3	80.3 ± 7.4	0.048*	59.7 ± 1.6	62.7 ± 9.1	0.750
ASV	HI/I	HI/I	–	HI/I	HI/I	–

*DTF* days to fifty percent flowering, *PH* plant height, *TN* Tiller number, *PN* Panicle number, *PL* Panicle length, *FL* flag leaf length, *FW* flag leaf width, *FS* number of filled spikelets, *TSP* total number of spikelets per plant, *SF* spikelet fertility, *GL* grain length, *GW* grain width, *HSW* hundred seed weight, *PY* plot yield, *TC* total carotenoids, *AC* amylose content, *GC* gel consistency, *ASV* alkali spreading value.

**Table 3:** Agronomic performance of GR2EIR64 at IRRI and GREBR29 at BRRI.

Traits	2015WS IRRI			2016DS IRRI			2016WS IRRI			2016 Boro		
	GR2E IR64	IR64	p value	GR2E IR64	IR64	p value	GR2E IR64	IR64	p value	GR2E BR29	BR29	p value
DTF	78.1 ± 0.3	81.0 ± 0.9	0.073	79.8 ± 0.2	80.0 ± 1.2	0.825	80.4 ± 0.2	82.3 ± 1.2	0.377	122.38 ± 0.2	124.3 ± 0.3	0.090
PH (cm)	118.6 ± 0.4	116.4 ± 1.2	0.285	96.4 ± 0.4	94.8 ± 2.5	0.535	115. 5 ± 0.5	120.5 ± 2.8	0.078	106.76 ± 0.4	107.9 ± 2.7	0.594
TN	13.8 ± 0.2	13.9 ± 0.5	0.525	14.3 ± 0.2	14.6 ± 1.2	0.825	14.1 ± 0.2	15.1 ± 1.2	0.377	-	-	-
PN	12.4 ± 0.2	12.3 ± 0.5	0.622	13.2 ± 0.2	13.7 ± 1.1	0.653	13.7 ± 0.08	13.5 ± 0.5	0.610	13.58 ± 1.0	12.93 ± 0.3	0.336
PL (cm)	27.0 ± 0.3	27.1 ± 0.1	0.460	24.7 ± 0.1	25.6 ± 0.7	0.232	27.3 ± 0.2	28.6 ± 1.0	0.215	26.59 ± 0.1	27.4 ± 0.9	0.210
FL (cm)	38.2 ± 0.3	34.2 ± 0.9	0.000	34.0 ± 0.4	33.8 ± 2.1	0.924	41.7 ± 0.4	40.9 ± 2.4	0.753	–	–	–
FW(cm)	1.7 ± 0.009	1.73 ± 0.02	0.389	1.5 ± 0.009	1.7 ± 0.05	0.001	1.6 ± 0.009	1.7 ± 0.05	0.233	–	–	–
FS	1045.1 ± 15.0	943.1 ± 46.2	0.068	1337.7 ± 22.8	898.4 ± 128.9	0.001	1110.0 ± 23.4	1045.0 ± 132.2	0.630	2147.61 ± 37.5	2022.15 ± 49.7	0.491
TSP	1751.4 ± 22.5	1580.9 ± 69.1	0.043	1679.2 ± 26.4	1510.9 ± 149.1	0.269	1593.9 ± 24.0	1596.9 ± 135.6	0.983	2603.29 ± 36.7	2574.99 ± 61.2	0.873
SF (%)	59.8 ± 0.5	59.6 ± 1.6	0.995	79.8 ± 0.7	59.5 ± 4.0	0.000	69.3 ± 0.9	65.1 ± 5.2	0.433	17.7 ± 0.5	21.47 ± 0.4	0.140
GL (mm)	9.6 ± 0.0.02	9.8 ± 0.04	0.015	9.7 ± 0.03	10.1 ± 0.2	0.069	10.0 ± 0.03	10.4 ± 0.2	0.027	8.43 ± 0.02	8.58 ± 0.09	0.134
GW (mm)	2.5 ± 0.007	2.4 ± 0.02	0.002	2.4 ± 0.008	2.4 ± 0.04	0.616	2.4 ± 0.007	2.4 ± 0.04	0.766	1.8 ± 0.005	1.81 ± 0.03	0.880
HSW (g)	2.5 ± 0.009	2.7 ± 0.03	0.003	2.6 ± 0.02	2.4 ± 0.09	0.069	2.6 ± 0.02	2.7 ± 0.09	0.323	2.81 ± 0.01	2.14 ± 0.05	0.289
PY (g)	297.0 ± 5.3	227.8 ± 16.4	0.062	757.6 ± 10.3	421.6 ± 58.3	0.000	638.1 ± 15.7	656.7 ± 88.8	0.837	3479.39 ± 0.03	3511.26 ± 0.01	0.864
TC (ppm)	4.4 ± 0.05	–	0.000	3.9 ± 0.04	–	0.000	4.4 ± 0.05	–	0.000	10.99 ± 0.55	–	–
AC (%)	19.4 ± 0.2	19.7 ± 0.9	0.785	22.1 ± 0.1	22.4 ± 0.9	0.528	19.5 ± 0.2	19.6 ± 1.4	0.956	24.0	27.0	0.004
GC	56.2 ± 1.1	74.7 ± 6.0	0.004	76.6 ± 1.1	69.7 ± 9.1	0.475	52.7 ± 1.1	68.3 ± 6.1	0.012	–	–	–
ASV	I	I		I	I	–	I	I	–	4.5	4.0	0.669

*DTF* days to fifty percent flowering, *PH* plant height, *TN* Tiller number, *PN* Panicle number, *PL* Panicle length, *FL* flag leaf length, *FW* flag leaf width, *FS* number of filled spikelets, *TSP* total number of spikelets per plant, *SF* spikelet fertility, *GL* grain length, *GW* grain width, *HSW* hundred seed weight, *PY* plot yield, *TC* total carotenoids, *AC* amylose content, *GC* gel consistency, *ASV* alkali spreading value.

### Correlation between yield, yield related traits and carotenoid content

The correlation among yield and yield related traits; and with total carotenoid content is presented in the Figs S6–S8. Over all there was no specific trend in correlations among different yield and yield related traits. Except in one environment carotenoid content was negatively but non-significantly associated with PY in all the three genetic backgrounds. The correlation analysis of carotenoid content between different seasons showed highly significant correlation in all the three genetic backgrounds.

### Effect of genetic background and environment on expression of carotenoids

**Figure 3:**
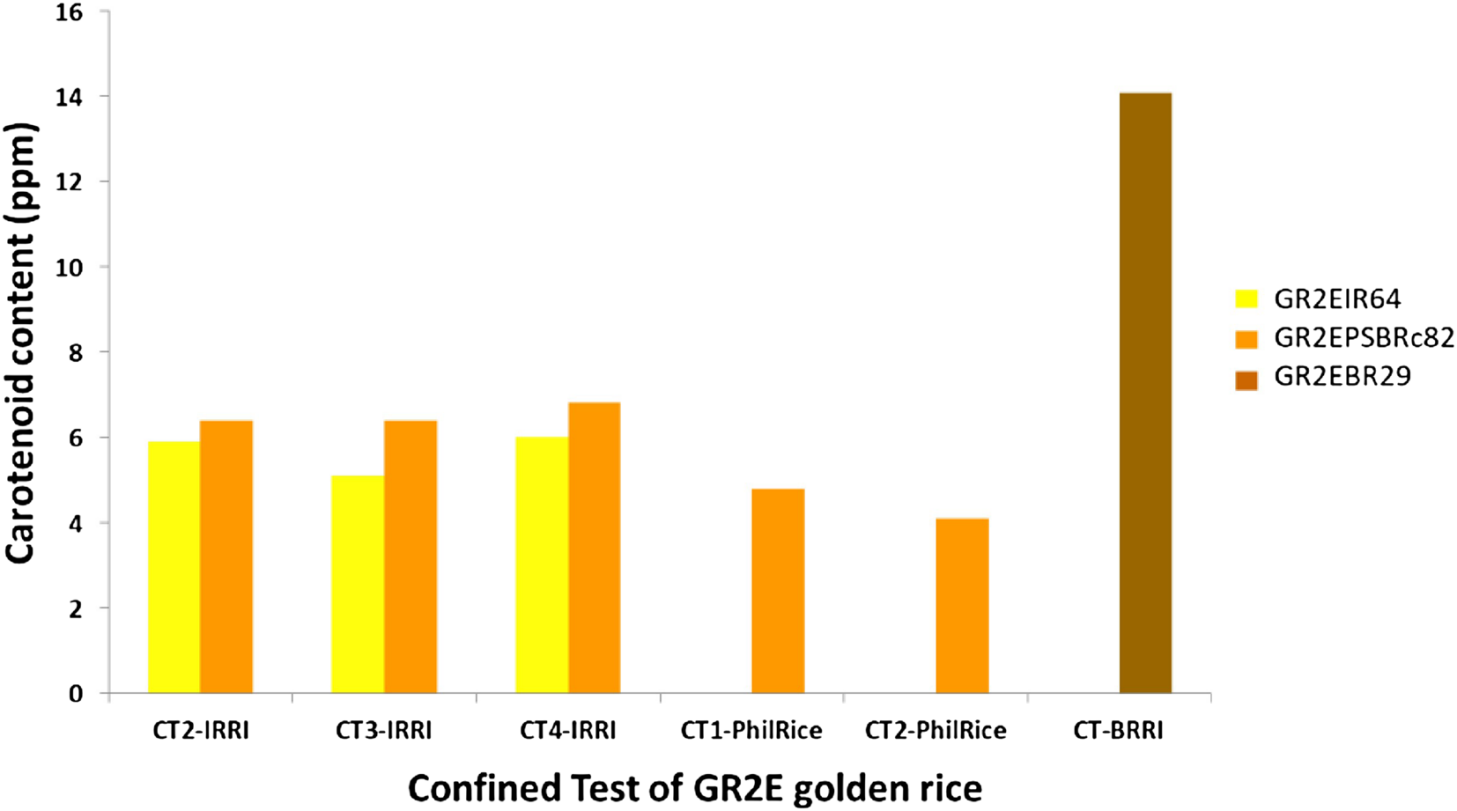
Carotenoid levels in different genetic backgrounds.

The combined analysis of variance for carotenoid content at two months after harvest showed that there were significant genotypic, seasonal and location effects on the expression of carotenoid content. However, there were no significant genotype and environmental interactions (G × E) for carotenoid content except CT2 PR vs CT4 (Table 4). However, among the three genetic backgrounds, expression of carotenoids was higher in GR2E BR29 ILs followed by PSBRc82 and lowest in GR2EIR64 ILs (Fig. 3, Fig S9). There were very highly positive significant correlations for carotenoid content estimated in different locations both within and between seasons (Figs S10–S12). In general carotenoids expression was bit higher in WS than in DS, but also among most of the CTs no significant G × E interaction was observed (Table 4).

**Table 4:** Analysis of variance and G × E for carotenoid content.

Trials	Genotype		Season or Location		Genotype × season or genotype × location	
	F value	p value	*χ*^2^ value	p value	*χ*^2^ value	p value
CT3 vs CT4 IRRI	9.526	0.001	2.788	0.095	2.271	0.131
CT2 PR vs CT3 IRRI	10.138	0.001	7.901	0.005	0.134	0.714
CT2 PR vs CT4 IRRI	6.289	0.001	7.298	0.007	10.308	0.001
Pooled (location effect)	41.228	0.001	9.315	0.002	0.182	0.669
Pooled (environment effect)	37.570	0.001	10.370	0.001	0.000	0.999

*CT* confined test, *IRRI* International Rice Research Institute, *PR* Philrice.

### Identification of superior GR2E NILs for multi-location evaluation

We selected five GR2E introgression lines each for PSBRc82 and IR64, for BR29 eight lines were selected from the CTs. These lines will be further evaluated in multi-location field testing in the Philippines and Bangladesh respectively. The list of selected lines and their corresponding agronomic performance is provided in Table 5. The ILs were similar to the respective recipient parents in all the agronomic, yield and yield traits measured, and the total carotenoids ranged from 3.8 to 5.5 ppm in the DS and 4.1 to 6.1 in the WS. Among the eight selected GR2E BR29 ILs no significant variation was observed in any trait except yield, with an advantage of 12.8% over BR29.

**Table 5:** Agronomic performance of GR2E lines selected for multi-location evaluation.

Designation	DTF		PH (cm)		GL (mm)		GW (mm)		HSW (g)		GY (kg/ha)		TC (ppm)		AC (%)	ASV	GC
	DS	WS	DS	WS	DS	WS	DS	WS	DS	WS	DS	WS	DS	WS			
IRRI																	
IR 112017 GR 2-E:23-30-98-B	78.3	92.0	94.6	134.5	8.9	8.9	2.4	2.5	2.4	2.4	6718.8	5749.8	4.7	5.2	19.8	HI	67
IR 112017 GR 2-E:23-40-51-B	80.0	88.7	96.3	136.6	9.1	9.4	2.5	2.5	2.5	2.6	5958.5	6280.6	4.8	5.0	22.0	I	40
IR 112019 GR 2-E:38-4-13-B	80.0	86.7	91.6	124.7	8.6	8.6	2.4	2.5	2.5	2.4	6305.0	5327.7	5.3	5.8	21.3	I	31
IR 112019 GR 2-E:38-4-27-B	79.7	87.0	91.1	120.8	8.8	9.0	2.4	2.4	2.4	2.4	6221.5	5110.6	5.5	6.0	17.9	HI/I	71
IR 112020 GR 2-E:57-18-75-B	79.3	93.3	94.9	133.5	9.1	9.3	2.4	2.5	2.3	2.5	6040.2	5900.3	5.1	4.9	19.2	HI	63
PSBRc82	78.3	87.0	98.5	136.2	9.6	9.6	2.4	2.5	2.6	2.5	6414.3	6859.9	0.6	0.5	17.4	HI/I	62
**PhilRice**																	
IR 112017 GR 2-E:23-30-98-B	80.7	86.3	95.4	127.5	8.9	9.2	2.5	2.4	2.3	2.3	9060.7	4737.4	3.9	5.1	17.4	I	71
IR 112017 GR 2-E:23-40-51-B	80.7	84.8	98.3	126.7	8.9	8.9	2.6	2.6	2.3	2.4	8484.4	5027.0	3.8	4.6	18.6	HI	63
IR 112019 GR 2-E:38-4-13-B	80.0	84.3	92.5	127.3	8.6	9.1	2.5	2.5	2.3	2.4	8541.5	4414.4	4.1	4.9	23.2	I	30
IR 112019 GR 2-E:38-4-27-B	81.3	86.4	90.7	127.8	8.7	9.1	2.5	2.4	2.1	2.4	8327.5	4276.4	5.1	6.1	23.0	HI/I	47
IR 112020 GR 2-E:57-18-75-B	80.7	85.8	98.6	133.1	9.2	9.1	2.5	2.5	2.3	2.5	8726.6	5737.3	4.2	4.1	18.1	HI	81
PSBRc82	81.3	85.9	98.3	129.2	9.5	9.3	2.5	2.5	2.6	2.5	8527.6	5061.2	0.6	0.3	19.3	HI	80
**BRRI**																	
IR 112060 GR2E:2-9-89-16-55	127.8		106.6		8.3		2.1		1.9		5700		13.8		25	–	–
IR 112060 GR2E:2-7-63-1-60	126.3		108.4		8.4		2.1		2.0		5950		12.3		23	–	–
IR 112060 GR2E:2-7-63-2-96	126.8		108.4		8.4		2.1		2.0		6373		12.1		24	4.5	–
IR 112060 GR2E:2-17-36-10-71	126.1		106.5		8.4		2.2		1.9		5751		13.3		23	–	–
IR 112062 GR2E:14-40-7-16-22	126.9		108.3		8.4		2.1		1.9		6287		13.2		23	–	–
IR 112062 GR2E:14-40-7-21-4	127.3		107.9		8.4		2.1		1.9		6178		12.8		25	–	–
IR 112062 GR2E:14-40-7-8-64	125.2		108.1		8.4		2.1		2.0		5865		12.0		23	–	–
IR 112062 GR2E:14-40-7-23-8	126.8		108.6		8.5		2.1		2.0		5670		12.7		24	–	–
BR29	125.5		109.0		8.5		2.2		2.0		5649		0.0		26	4.0	–

*DTF* days to fifty percent flowering, *PH* plant height, *TN* Tiller number, *PN* Panicle number, *PL* Panicle length, *FL* flag leaf length, *FW* flag leaf width, *FS* number of filled spikelets, *TSP* total number of spikelets per plant, *SF* spikelet fertility, *GL* grain length, *GW* grain width, *HSW* hundred seed weight, *PY* plot yield, *TC* total carotenoids, *AC* amylose content, *GC* gel consistency, *ASV* alkali spreading value, *DS* dry season, *WS* wet season.

## Discussion

Most of the dietary vitamin A is of plant origin in the form of provitamin A that is converted to vitamin A in the body^17^. VAD is persistent in most of the rice eating countries in Asia, Africa and Latin America^18,19^. Therefore, enriching rice with provitamin A through biofortification is a viable and complementary intervention to tackle the VAD. The provitamin A trait was introduced into the rice variety Kaybonnet through genetic engineering^13^, which has a temperate *japonica* genetic background and is not well adapted to the tropical conditions in most rice growing Asian countries. We developed GR2E event introgressed golden rice ILs in the genetic backgrounds of IR64, PSBRc82 and BR29.

### Introgression of the GR2E produced agronomically superior plants

Golden rice GR2E is genetically stable and molecularly clean event useful for breeding (https://www.dropbox.com/sh/qpiz0cftefcaceq/AAByIpj_HED3zgqH7ufW7A-ta?dl=0; https://www.foodstandards.gov.au/code/applications/Documents/A1138%20Application_Redacted.pdf). The breeding process to develop GR2E introgression lines did not show any abnormal plant phenotypes both in homozygous and hemizygous conditions indicating the genetic stability of the GR2E gene and trait expression. Both the phenotypic and genotypic based segregation analysis showed typical Mendelian segregation ratio in different segregating generations. GR2E advance backcross progenies were phenotypically very similar to their respective recipient parents. Transgenic events with single copy, clean integration and showing normal Mendelian segregation are considered ideal for research and breeding purposes, as they do not alter the host plant genome^20–22^.

Agronomic performance at field level and G × E studies showed that the GR2E gene did not alter any of the traits of the recipient parents in all its zygosity conditions. Overall plant performance was better during DS and among the genetic backgrounds the GR2EPSBRc82 lines performed better than the GR2EIR64 lines. Morphological traits such as panicle type, panicle exertion, grain shape, flag leaf length and width were similar for the GR2E ILs. Many lines performed equally similar to the respective recurrent parents, allowing the selection of advanced lines in all backgrounds for further testing in multi-location trials. The results showed that back cross process recovered almost all the desirable agronomic, yield and grain quality traits of the respective parents with significant expression of vitamin A. Despite many typhoons, heavy rains and high winds during the trials. There were no severe lodging incidences observed. Insects and diseases incidences were monitored during the two growing seasons at two different plant growth stages: maximum tillering stage (vegetative stage) and 50% flowering. Generally, crop stand was good with manageable level of insect pests and diseases during the growing seasons. Insects observed (both pest and beneficial insects) were found to be present in both test materials. We did not notice any difference between GR2E introgression lines and their respective recipient parents for the pest or diseases pressure on the crop across the confined field tests.

Woodfield and White^23^, and Badenhorst et al*.*^24^ opined that development of transgenic product is not limited only to transformation, but also includes breeding through further backcrossing of transgenes with recipient parents and selection for desired traits of interest, in order to expedite commercial product development. For commercial deployment of any new variety with one or more introduced new trait(s) of a staple crop, in parallel to yield and other key agronomic traits, the newly developed variety should have essentially similar or better performance against biotic and abiotic stresses and grain quality traits compared to recipient variety; the introduced trait(s) should not alter these traits of the recipient variety^25,26^.

### Grain quality and proximate composition of GR is similar to recipient rice varieties

Furthermore, different cooking and eating quality traits like, AC and ASV did not show any significant difference between the ILs and their respective recipient parents in any CTs. The golden rice breeding lines with significant amount of provitamin A accumulated in the grains helps to tackle VAD in high risk countries such as Bangladesh and the Philippines. However, it is a requirement to assess the composition of genetically modified crops to see if any significant changes in grain quality, nutrients and anti-nutrients contents in comparison to traditional counterpart and to assess the safety of the intended or unintended changes^27,28^. The compositional analysis of golden rice showed that all the compounds measured are within the biologically acceptable range and does not pose any risk to human health^29^. Earlier reports on transgenic products for insect and herbicide tolerance have also shown that little biologically meaningful changes in grain quality, nutrient and anti-nutrient composition^30^. There was a clear environmental effect, even though total carotenoids varied with environments, the genotypes with high carotenoids were always the best in all the locations. Such variations in trait expression due to environmental and agronomic factors and genetic basis have been well explained^31,32^.

### Genetic background and environment influences carotenoid expression

Stable trait expression and minimal G × E for any trait of importance, especially for grain micronutrients and vitamins is essential for varietal release as well as for their successful adoption^4,33,34^. Total carotenoids were well correlated across the sites and generations; and expressed stably across the environments but there is a genetic background effect. Carotenoids expression varied even within segregating lines of different generations in each of the genetic backgrounds. So targeted breeding and careful selection of progenies with carotenoids test in each generation is necessary for advancing the lines. Mapping background QTLs and genes and using them in MAB can provide opportunity for precise development of GR lines with highest expression. The carotenoid levels were found to vary across the genetic backgrounds, locations and seasons but there were no significant G × E interactions. The highest expression of carotenoids was observed in BR29 background and the lowest in IR64 background. Several earlier attempts to develop golden rice events and introgression lines had to face the genetic background effects. Transgenic events developed in the *indica* backgrounds of IR64 and BR29 reported lower expression of GR genes in IR64 and higher expression in BR29 transformants, even ILs developed in IR64 showed lesser expression^35^. Moreover, ILs did not show any significant difference in yield when expressing the genes in the carotenoid pathway^36^. In our study also lowest expression was noticed in IR64. Simultaneously efforts are being made to develop next generation golden rice events with elevated levels of carotenoids with longer stability^37–39^. However, a genetic background effect is still a major bottle neck for introgression of carotenoid trait. Background effect on the expression of introduced traits was reported in rice for submergence tolerance, yield and related traits, disease resistance and drought tolerance^15,16,40,41^.

The variation in carotenoid concentration in grains might be due to variations in sunlight exposure and intensity across the locations and seasons^42^. Differential accumulation of β-carotene due to variation in exposure period and intensity of sunlight was also observed in algae, carrots, pumpkin and maize^43–46^. Moreover, like other carotenoids containing crops the carotenoid concentration in the grains of golden rice degrades over time after harvest. The degradation rate is very high at first few weeks after harvest and it becomes very slow after 6–8 weeks (data not shown). The carotenoids degradation rate is highly influenced by the storage temperature, moisture and exposure to light of the storage environment^22,47^. So, development of golden rice varieties with stable carotenoids expression is essential to achieve the impact^37^. However, there might be genotypic effect on the retention ability for carotenoids in rice grain. Understanding background effect and standardization of post-harvest handling is needed to achieve desired level of carotenoids in the introgression lines of multiple backgrounds.

### Superior introgression lines were identified for multi-location trials

The five back crosses of GR2E gene into three genetic backgrounds resulted in identification of ILs similar to respective recipient parents. Adoption by the farmers and preference by the consumers for a specific crop variety particularly rice introduced with a new trait largely depends on its yield, grain quality and eating quality parameters. The introduced trait should be stable over locations and seasons to expedite the adoption level. Considering the present levels of carotenoids and per capita consumption in these target countries, the resulting ILs would be able to supply 30–50% of the EAR for vitamin A for the high risk population group if GR2E rice is consumed regularly.

## Materials and methods

### Development of GR2E near isogenic lines

**Figure 4:**
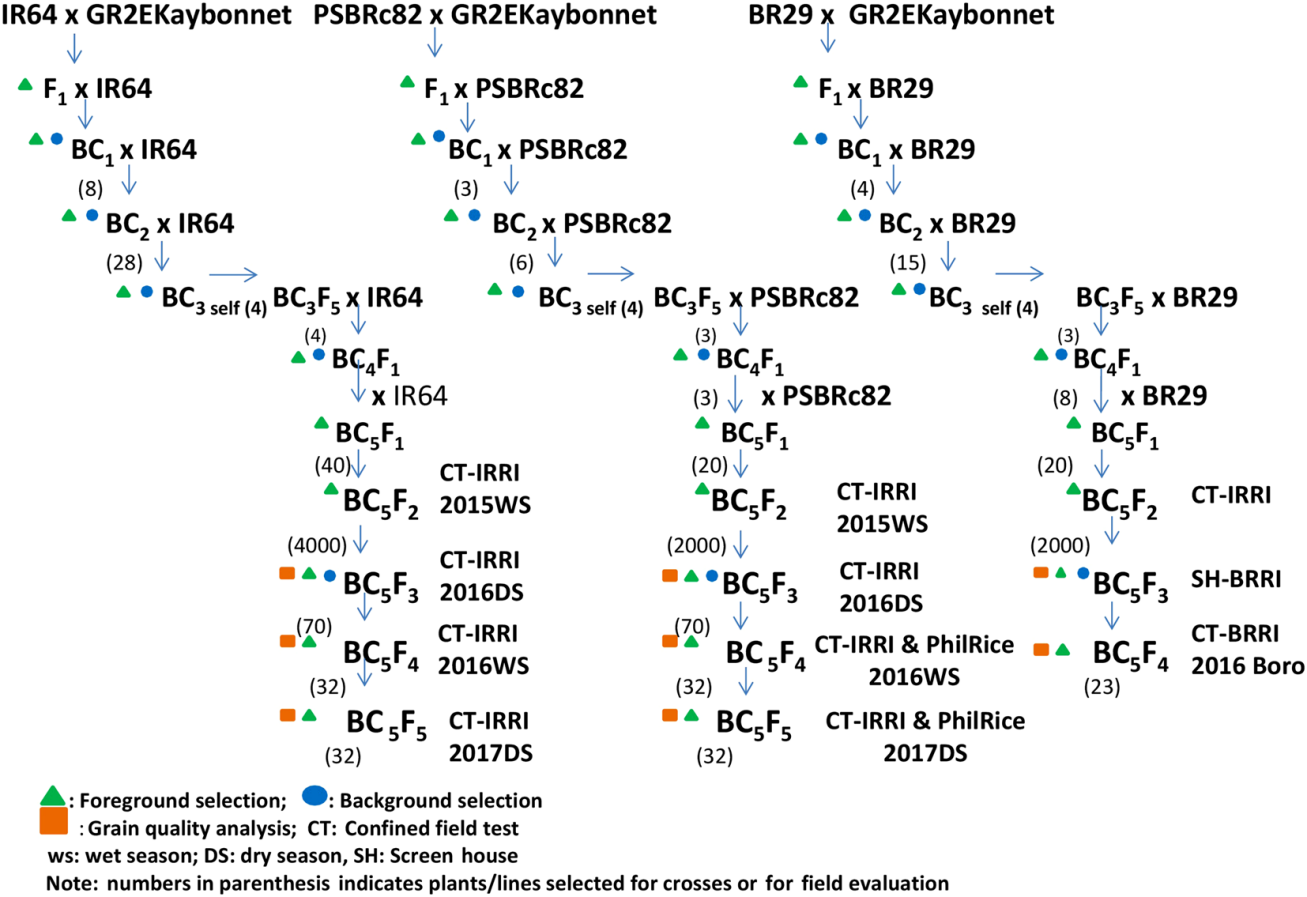
Development and evaluation of GR2E introgression lines.

Kaybonnet is a high yielding japonica rice variety with blast resistance and excellent milling quality commercially cultivated in the USA. The genetic modification was made by the addition of two genes, phytoene synthase (Zmpsy1) from *Zea mays* and carotene desaturase (crtI) gene from the common soil bacterium, *Pantoea ananatis* (syn. *Erwinia uredovora*). The GR2E Kaybonnet was crossed with the popular high yielding and adopted rice varieties such as IR64, PSBRc82, and BR29. IR64 is popular in most of the Asian countries, PSBRc82 in the Philippines, and BR29 in Bangladesh. In each generation, segregating materials were genotyped using GR2E event specific molecular marker. Plants containing the GR2E event and phenotypically similar to respective recipients were selected and backcrossed in each backcross generation to advance the materials to BC_5_F_2_. Background selections were performed using 100 randomly selected SSR markers in BC_1_ and BC_2_, while selected plants from BC_3_, BC_4_ and BC_5_ were genotyped using the 6 K SNPs set at Genotyping Service Laboratory, IRRI. Only yellow-colored BC_5_F_2_ seeds were separated and analyzed for total carotenoid content. A total of 40 BC_5_F_2_ families for IR64 and 20 families each for PSBRc82 and BR29 were selected for evaluation in the confined test at IRRI. We have provided details of MAB scheme and evaluation of introgression lines in the Fig. 4.

### Experimental materials used in the confined tests

A total of 8000 individual plants comprised of 4000 BC_5_F_2_ plants from GR2E IR64, 2000 plants each from GR2E PSBRc82 and GR2E BR29 were included in a CT in the dry season of 2015 (2015DS). Plants were genotyped using GR2E specific markers and homozygous plants were selected. Selected BC_5_F_3_ homozygous plants from each genetic background along with the respective recipient and donor parents were evaluated in a series of CTs at IRRI and PhilRice in the Philippines and at BRRI in Bangladesh. The list of GR2E materials evaluated and the details of the CTs is provided in the Supplementary Table S1. Three CTs were conducted for GR2E IR64 and GR2E PSBRc82 at IRRI, while the selected lines of GR2E PSBRc82 were evaluated for two seasons at PhilRice. Further, BC_5_F_3_ seeds of GR2E BR29 were sent to Bangladesh, multiplied in the screen house, and further evaluated in a CT at BRRI, Gazipur, for one season in 2016.

### Crop management and observations

Seeds of the selected plants of GR2E introgression lines, recipient and donor parents were seeded in trays. Seedlings were transplanted at 21 days after sowing with a standard spacing of 20 × 20 cm. Details of the experimental design and layout are provided in Tables S1 and S2. Standard agronomic practices were followed to raise a good crop, including the application of need-based plant protection measures to protect the crop from diseases and insect pests. Data were gathered on key agronomic, yield and yield-related traits; and total carotenoid content was measured two months after harvest. Grain quality data were generated from the selected lines of CT2 and from all lines included in CT3 and CT4. Insect pest infestations and disease incidences were recorded at maximum tillering and at 50% flowering. Agronomic traits were measured on five random plants from each entry. Days to 50% flowering was recorded on a whole plot basis. At maturity, five selected plants were harvested from individual plots and the remaining inner plants were harvested in bulk. Final plot yield was adjusted to a uniform grain moisture content of 14%.

### Genotyping

DNA was extracted using fresh leaf samples and following a modified cetyl trimethylammonium bromide (CTAB) protocol^48^. Nanopore was used to check the quality and quantity of the DNA extracted. The DNA samples were diluted with distilled water into an equal concentration of 25 ng/µl. Amplification of event specific markers using polymerase chain reaction (PCR) was carried out with a 10 µl reaction mixture that contained 1.5 µl of DNA template, 1.0 µl of 10 × PCR buffer with MgCl_2_, 0.5 µl each of forward and reverse primers, 0.2 µl of 1 mM dNTP and 0.1 µl of Taq DNA polymerase and 5.7 µl distilled water. The amplification reaction was carried out in a 96-well PCR plate in a thermocycler using the following temperature profile: denaturation, 95 °C for 5 min; 35 cycles of denaturation at 95 °C for 45 s, annealing at 55 °C for 45 s and extension at 72 °C for 45 s; and final extension at 72 °C for 8 min and long-term storage at 10 °C. Amplification products were separated by gel electrophoresis on 1.2% agarose (0.5 × TBE; 160 V for 45 min) and visualized using SYBR Safe DNA stain and imaging using an AlphaImager HP (Protein Simple, San Jose, CA) gel documentation system. The GR2E specific primer sequences as follows.

- ZD-E1-P1 5′-GCTTAAACCGGGTGAATCAGCGTTT-3′
- ZD-E1-P2 5′-CGAGAGGAAGGGAAGAGAGGCCACCAA-3′
- ZD-E1-P3 5′-CTCCCTCACTGGATTCCTGCTACCCATAGTAT-3′

### Grain quality analysis

Grain quality analysis was carried out at the Analytical Service Laboratory (ASL) of IRRI. We measured/analyzed grain length and width, amylose content, alkali spreading value and gel consistency, using standard protocols^49^. Similar analyses were performed at BRRI on grain samples of GR2E BR29.

#### Amylose content

Amylose content (AC) was determined on milled rice extracts using a segmented flow analyzer. Rice samples were ground to a fine powder using a cyclone mill. Sodium Hydroxide and Ethanol were added to a test portion of the sample and heated in a boiling bath for 10 min. Acetic acid and Iodine solution was mixed with the aliquot of the test solution to form a blue starch iodine complex and its absorbance was measured at 620 nm using a colorimeter^49^. The result of the analysis was reported as apparent amylose to take into account the contribution of amylopectin present in the rice, which also forms a blue color starch iodine complex.

#### Gelatinization temperature

Rice starch gelatinization temperature (GT) was estimated by determining the alkali spreading value (ASV) of milled rice grains in potassium hydroxide solution. Six kernels of whole milled rice were incubated with 10 ml of 1.7% KOH for 23 h at ambient temperature (25 °C). The appearance and disintegration of the endosperm was visually rated depending on the intensity of spreading and swelling. ASV of 1–2 was classified as high GT, 3 for intermediate to high GT, 4–5 for intermediate GT and 6–7 for low GT.

#### Gel consistency

Samples of milled rice were ground to a fine powder, placed in a culture tube and suspended in a mixture of ethanol and 0.2 N KOH containing thymol blue and incubated in a boiling water bath for 15 min, followed by cooling to room temperature (15 min) and placing in an ice bath (20 min). Gel consistency of the rice paste (4.4% w/v) was determined by measuring the length of the cold gel in the culture tube after placing horizontally for 1 h. Rice was differentiated into three consistency types—soft (61 to 100 mm), medium (41 to 60 mm) and hard (27 to 40 mm).

#### Carotenoid concentrations

Total carotenoid concentration was estimated following the protocol developed by Gemmecker et al*.*^50^. Dehulled and polished rice seeds were ground to a fine powder using a modified paint shaker and accurately weighed amounts (ca. 500 mg) were dispensed into 15-ml Falcon tubes, mixed by sonication with 2 ml distilled water and incubated for 10 min at 60 °C. Cooled samples were centrifuged (3000*g*, 5 min) and the supernatant fractions were transferred to new 15-ml tubes. Acetone (2 ml) and 100 μl of the lipophilic metallo organic dye, VIS682A (20 μg/ml; QCR Solutions Corp.), as an internal standard were added to each sample followed by mixing with short pulses of sonication and centrifugation (3000*g*, 5 min). Supernatants were transferred to 15-ml tubes and the pellets were re-extracted twice more with 2-ml volumes of acetone and the resulting supernatant fractions were combined. Two ml petroleum ether (PE): di-ethyl ether (DE) (2:1 v/v) was added to each combined supernatant fraction (ca. 8 ml) and volumes were adjusted to 14 ml with distilled water. After vortexing, phase separation was achieved by centrifugation (3000*g*, 5 min). The organic phase was recovered by pipetting out and transferred into a 2 ml graduated Eppendorf tube and the remaining aqueous phase was re-extracted with another 2 ml PE:DE (2:1 v/v), followed by centrifugation (3000*g*, 5 min). The combined organic phases were dried using a vacuum-concentrator (Eppendorf concentrator 5301) and re-dissolved in 1 ml acetone. Maximum absorbance of sample extract at 450 nm and maximum absorbance of internal standard at 680 nm was determined using DU730 Beckman Coulter UV/VIS spectrophotometer. Concentrations of total carotenoids were determined from A450 nm assuming an average E450  nm = 142, 180 l mol^−1^ cm^−1^ in acetone using the Beer-Lambert law corrected for sample dilution and normalized to the internal standard.

### Statistical analysis

All statistical analyses were performed as a linear mixed model using R^51^ and PB Tools v1.0^52^.

Mixed model for single site analysis:

$${\text{yij }} = \, \upmu {\text{i }} + {\text{ bj }} + {\text{ eij}}$$

where µi denotes the mean of the ith entry (fixed effect), bj denotes the effect of the jth block, and eij denotes the residual error.

Mixed model for multiple site analysis:

$${\text{yijk }} = \, \upmu {\text{i }} + {\text{ lk }} + {\text{ bj}}\left( {\text{k}} \right) \, + \, \left( {\upmu {\text{l}}} \right){\text{ik }} + {\text{ eijk}}$$

where µi denotes the mean of the ith entry (fixed effect), lk denotes the effect of the kth site, bj(k) denotes the effect of the jth block within the kth site, (µl)ik denotes the interaction between the entries and sites (random effect), and eijk denotes the residual error.

### Mean comparison and correlation analysis

The differences in least square (LS)-mean values between GR2E rice and the control rice were tested at first step followed by significant difference (p < 0.05) was identified in the multi-year combined-sites analysis^53^. Correlation among different traits from all the replicated trials was carried out using R Program^51^.

## Supplementary Information

Supplementary Information.
