# Supplementary material for: Development and characterization of GR2E Golden rice introgression lines
Source: Sci Rep. 2021 Jan 28;11:2496. doi: 10.1038/s41598-021-82001-0 (PMC7843986; doi:10.1038/s41598-021-82001-0)
Supplement: Supplementary file 1 — Supplementary Information. [file 41598_2021_82001_MOESM1_ESM.docx]

**Development and characterization of GR2E Golden rice Introgression lines**

B.P. Mallikarjuna Swamy^1*^, Severino Marundan Jr.^1^, Mercy Samia^1^, Reynante L Ordonio^2^, Democrito B. Rebong^2^, Ronalyn Miranda^2^, Anielyn Alibuyog^2^, Anna Theresa Rebong^2^, Ma. Angela Tabil^2^, Roel R. Suralta^2^, Antonio A. Alfonso^2^, Partha Sarathi Biswas^3^, Md. Abdul Kader^3^, Russell F. Reinke^1^, Raul Boncodin^1^, and Donald J. MacKenzie^4^

^1^International Rice Research Institute (IRRI), DAPO Box 7777, Metro Manila, Philippines

^2^Philippines Rice Research Institute (PhilRice), Maligaya, Science City of Munoz, Philippines

^3^Plant Breeding Division, Bangladesh Rice Research Institute (BRRI), Gazipur, Bangladesh

^4^Institute for International Crop Improvement, Donald Danforth Plant Science Center, Saint Louis, MO, USA

**Table S1. Details of confined field tests conducted at IRRI and PhilRice**

**Activity**

**CT2-IRRI**

**CT3-IRRI**

**CT4-IRRI**

**CT1-PhilRice**

**CT2-PhilRice**

**CT-BRRI**

70 GR2EPSBRc82

32 GR2EPSBRc82

32 GR2EPSBRc82

70

GR2EPSBRc82

32 GR2EPSBRc82

23 GR2EBR29

70 GR2EIR64

32GR2EIR64

32 GR2EIR64

Nulls and

Parental lines

Nulls and Parental

lines

Nulls and Parental

lines

Nulls and Parental lines

Nulls and Parental lines

Nulls and Parental lines

Experimental design

P-rep

RCBD with 3 reps

RCBD with 3 reps

P-rep

RCBD with 3 reps

RCBD with 3 reps

Sowing

May 29, 2015

Nov 17, 2015

Apr 20, 2016

June 5, 2015

Nov 26, 2015

Dec 20, 2015

Seedling pulling

June 17, 2015

Dec 7, 2015

May 10, 2016

June 21,2015

Dec 15, 2015

14-Feb-16

Transplanting

June 18, 2015

Dec 8, 2015

May 10, 2016

June 23, 2015

Dec 17, 2015

14-Feb-16

Harvesting

Sept 21,22, 28 and 29, 2015

March 10 & 11, 2016

Aug 12 & 19, 2016

Sept 30 - Oct 8,

2015

March 15 and 16,

2016

9 May- 21 May,

2016

Experimental

**Table S2. Details of P-Rep design**

| **Lines** | **Genetic background and location** | | |
| --- | --- | --- | --- |
|  | **IR64 - IRRI** | **PSBRc82 - IRRI** | **PSBRc82 -Philrice** |
| IR64 parent | 14 reps |  |  |
| PSBRc82 parent |  | 14 reps | 14 |
| Kaybonnet wildtype | 14 reps | 14 reps | 14 |
| Kaybonnet GR2-E | 14 reps | 14 reps | 14 |
| Nulls | 15 lines 7 reps | 15 lines 7 reps | 15 lines 7 reps |
| Homozygous lines | 5 lines 7 reps | 9 lines 7 reps |  |
| Homozygous lines | 22 lines 6 reps | 9 lines 6 reps |  |
| Homozygous lines | 10 lines 5 reps | 16 lines 5 reps |  |
| Homozygous lines | 8 lines 4 reps | 6 lines 4 reps |  |
| Homozygous lines | 5 lines 3 reps | 12 lines 3 reps |  |
| Homozygous lines | 9 lines 2 reps | 11 lines 2 reps |  |
| Homozygous lines | 19 lines 1 rep | 21 lines 1 rep |  |

**
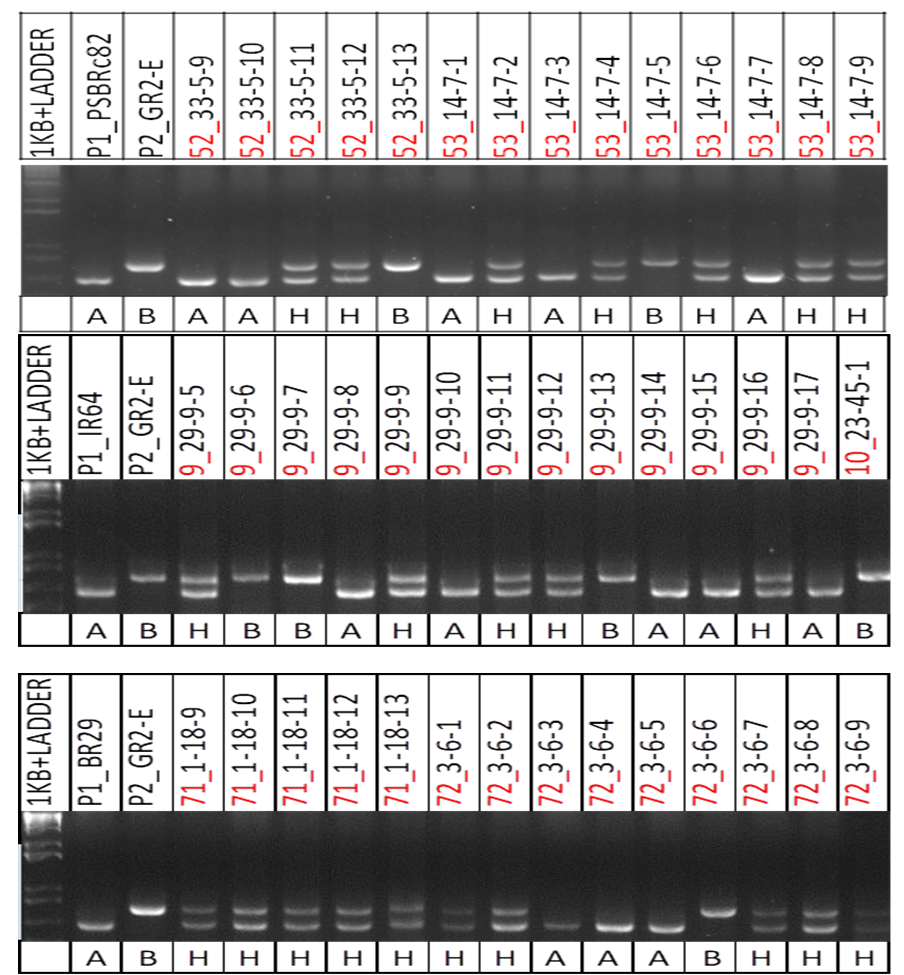
**

Fig S1. Segregation analysis of GR2E


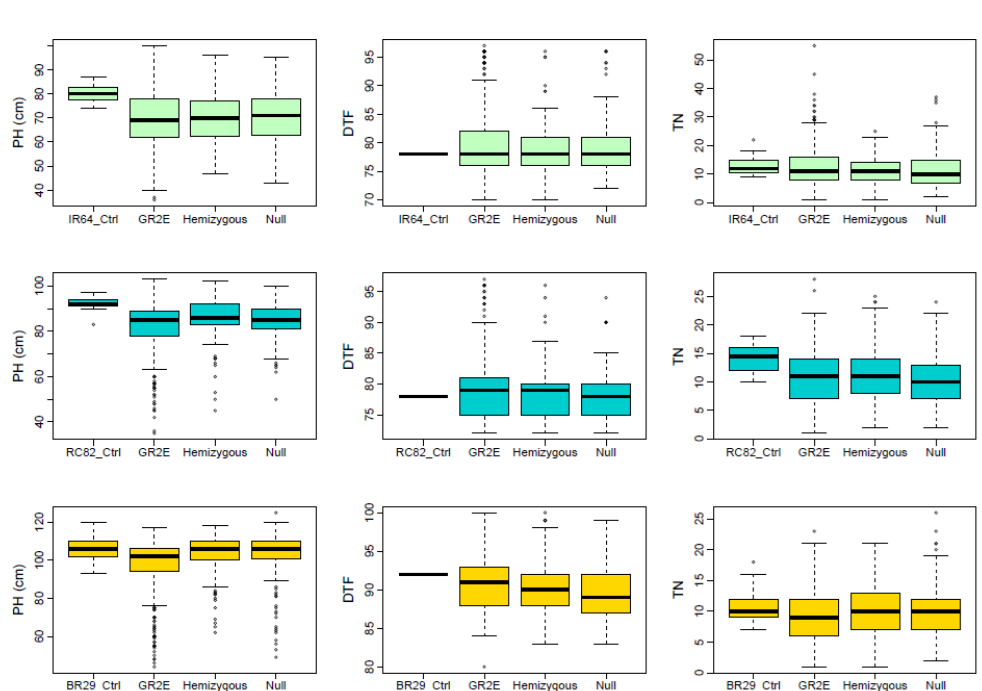


Fig S2. Box plot showing distribution of traits in different zygosity classes of GR2E


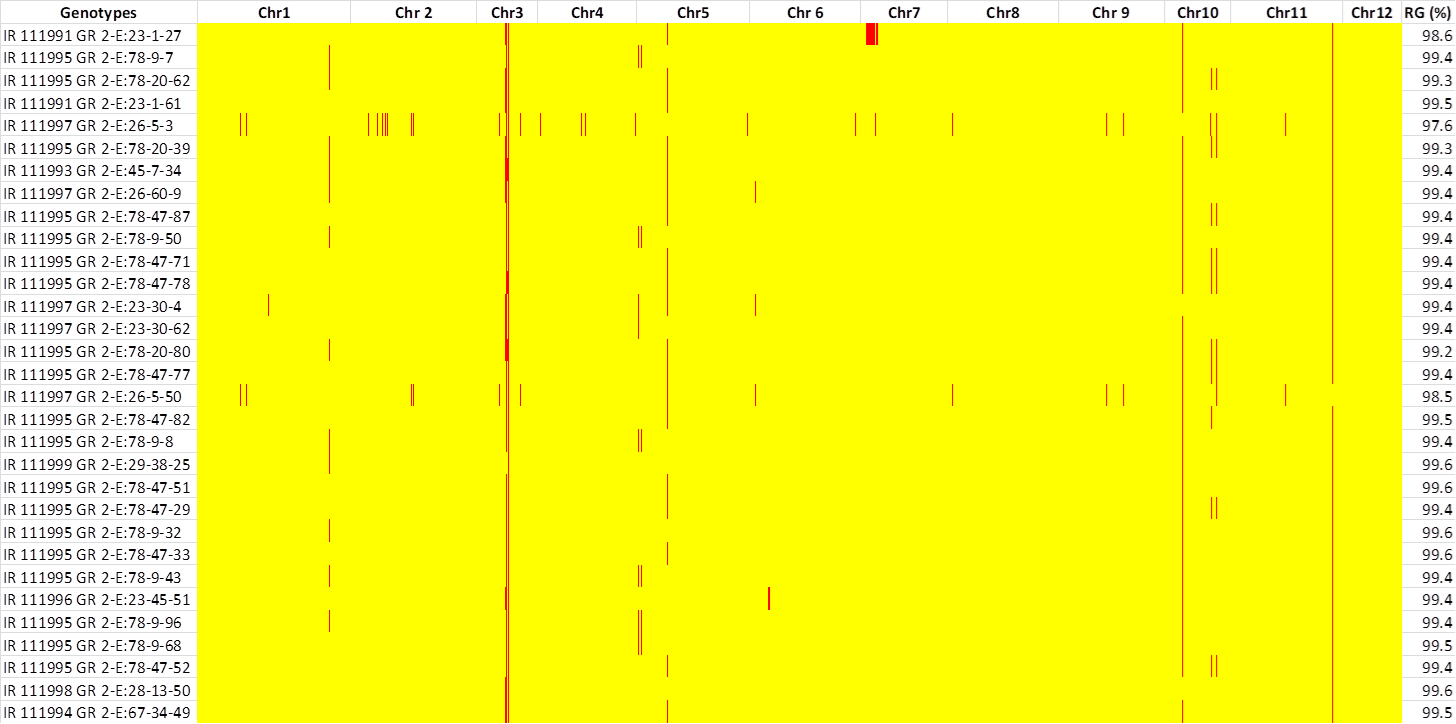


Fig S3. Graphical genotype of GR2E IR64 Introgression lines showing background recovery

Note: Red bars indicate GR2E (donor segments), RG: Recipient Parent genome


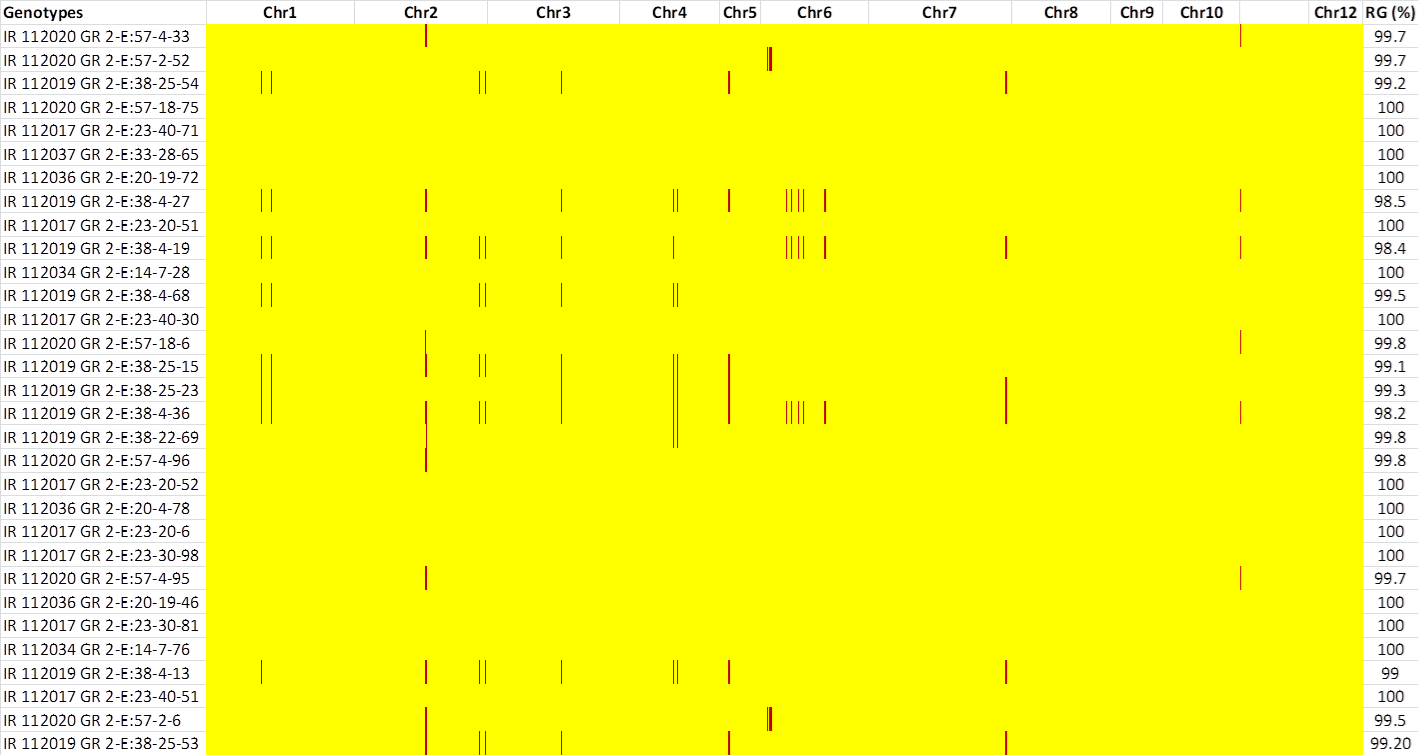


Fig S4. Graphical genotype of GR2E PSBRc82 Introgression lines showing back ground recovery

Note: Red bars indicate GR2E (donor segments), RG: Recipient Parent genome


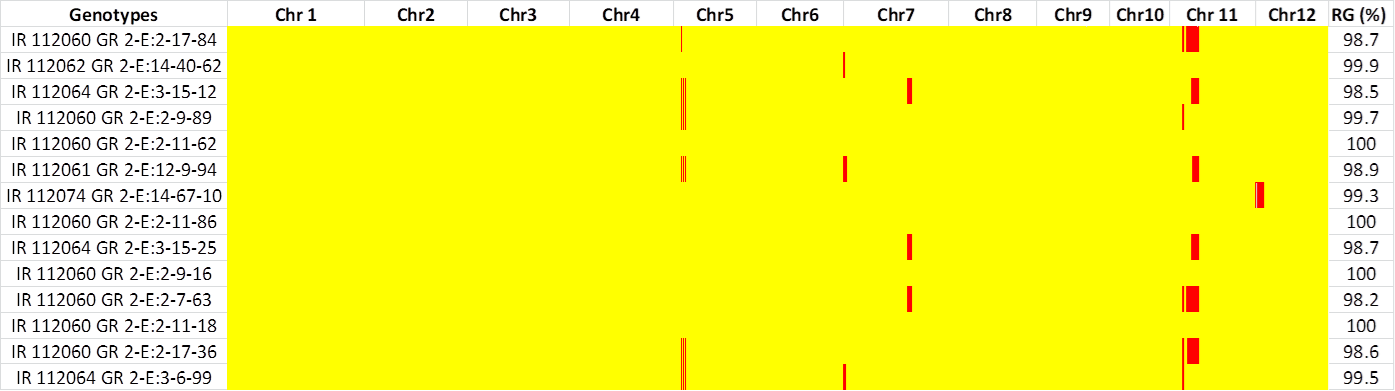


Fig S5. Graphical genotype of GR2E BR29 Introgression lines showing back ground recovery

Note: Red bars indicate GR2E (donor segments), RG: Recipient Parent genome


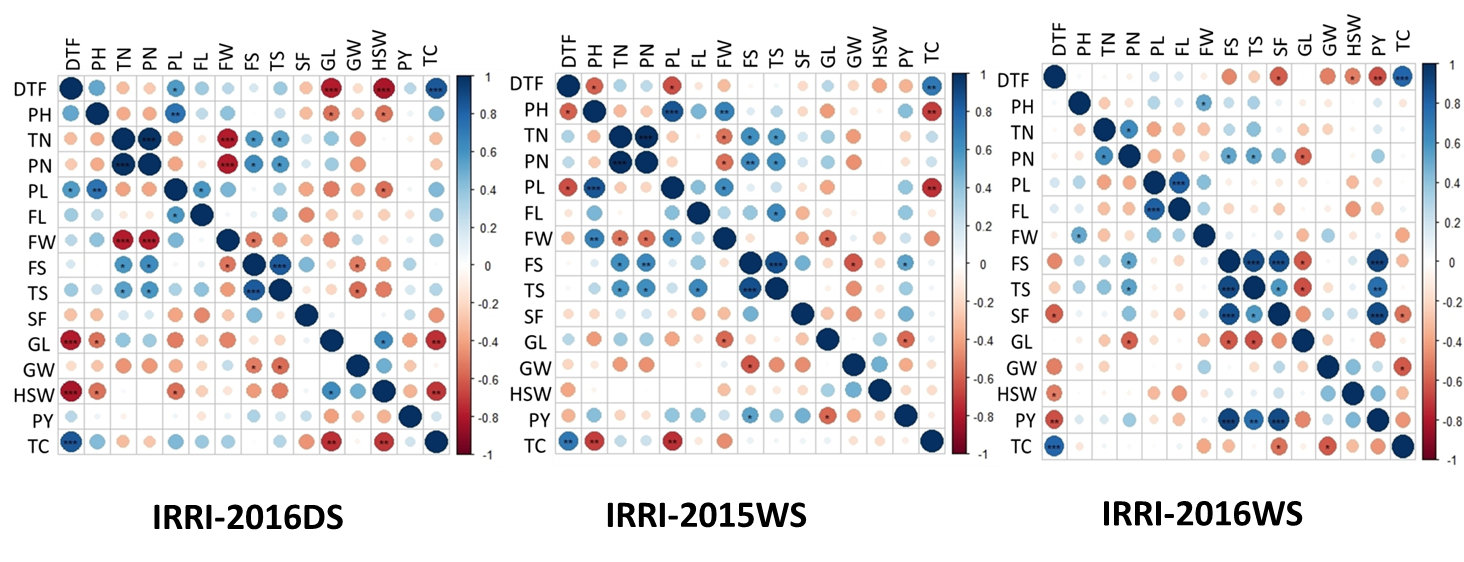


Fig S6. Correlation among different traits measured in GR2E IR64 in different seasons at IRRI


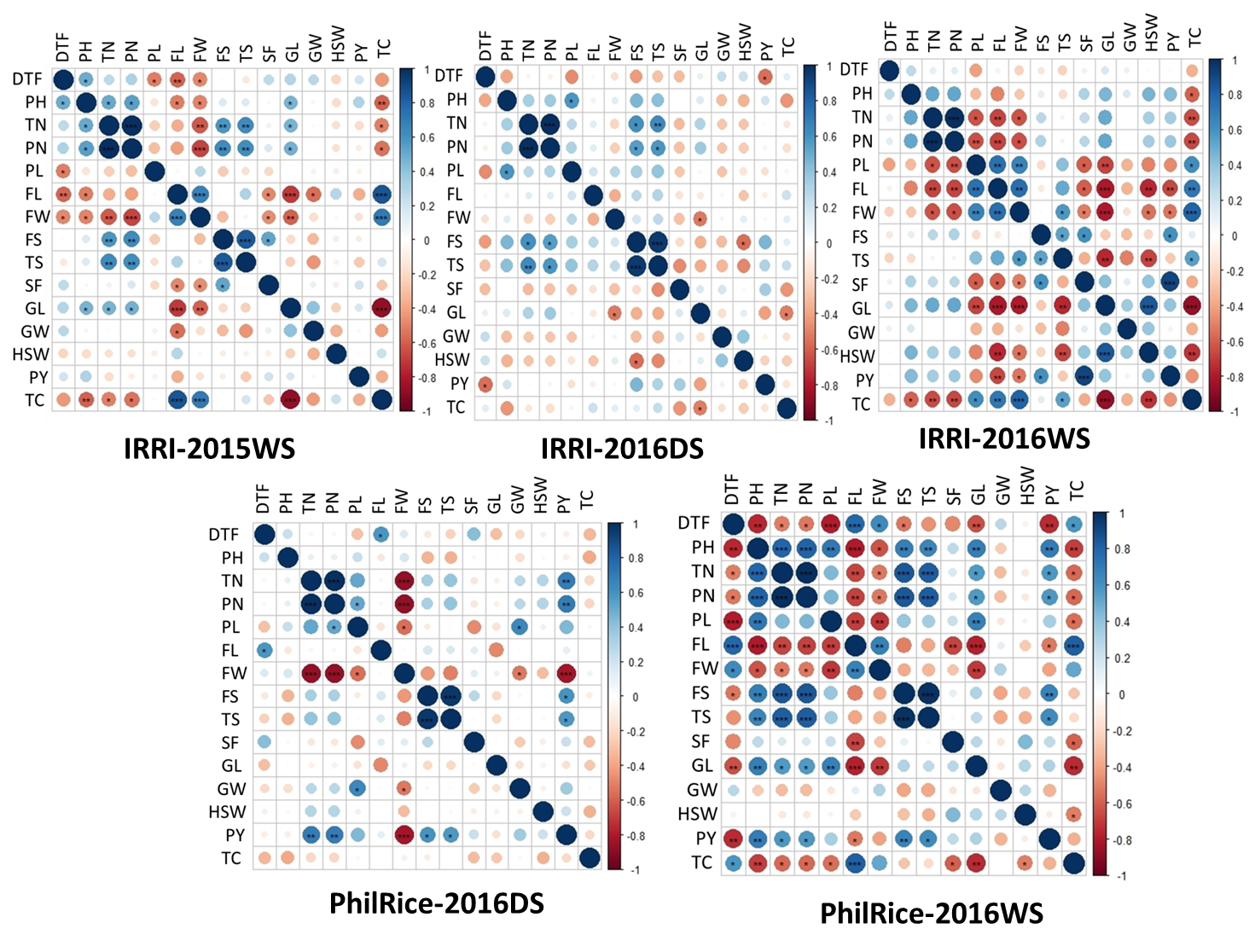


Fig S7. Correlation among different traits measured in GR2E PSBRc82 during different seasons and locations

**
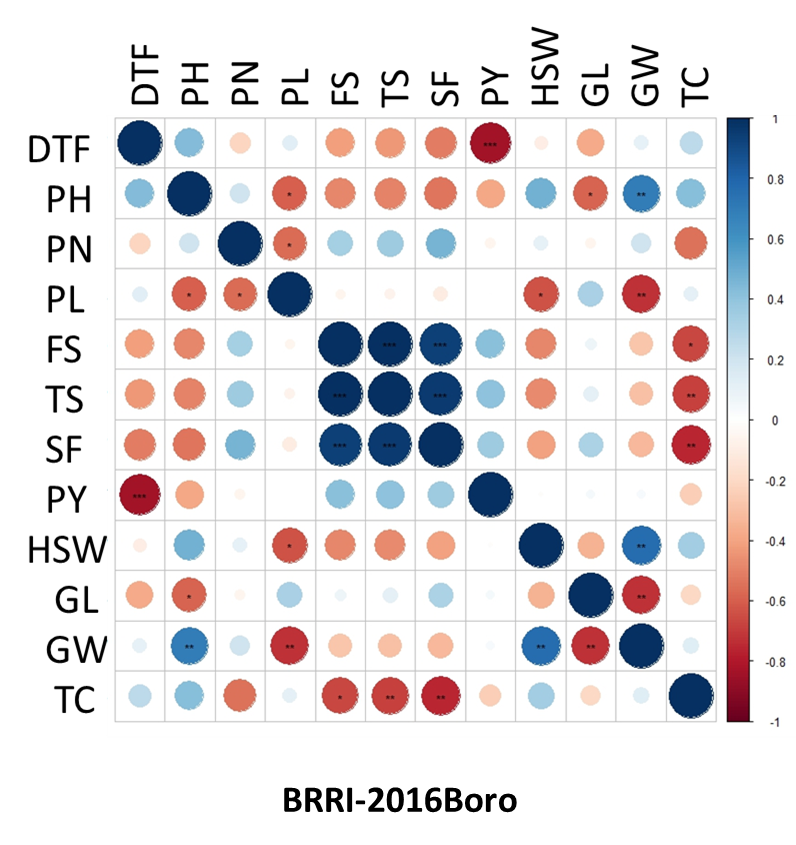
**

Fig S8. Correlation among different traits measured in GR2EBR29 at BRRI.

**
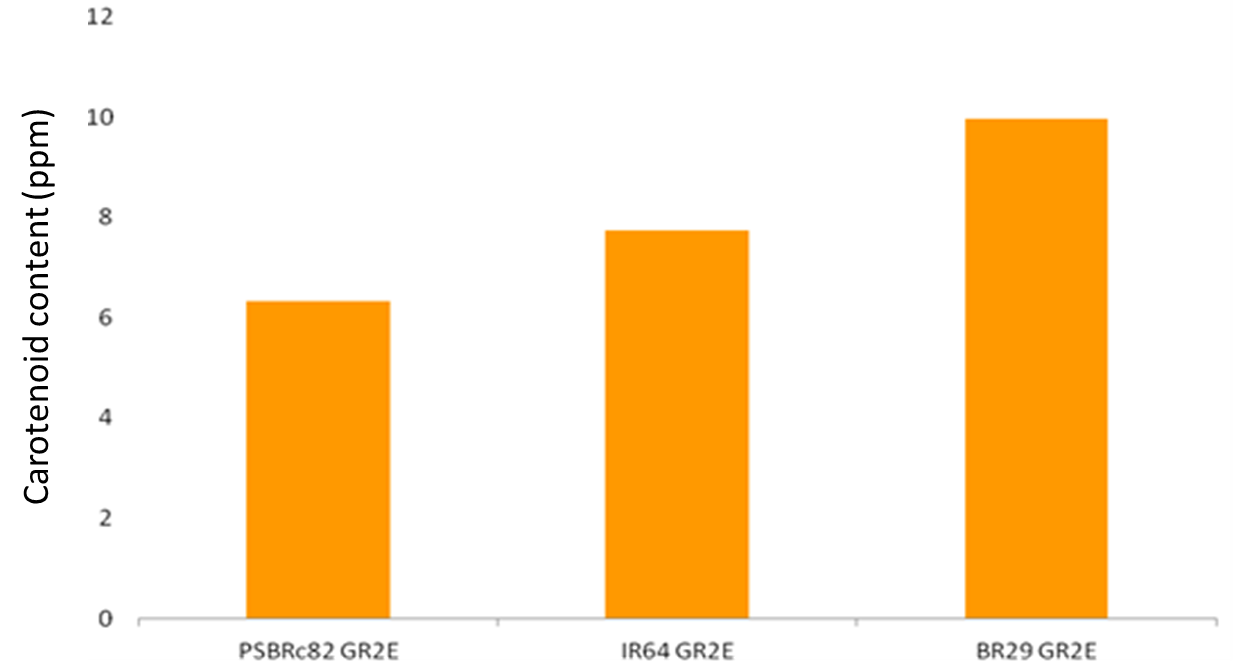
**

Fig S9. Carotenoid levels in three different genetic backgrounds during 2015WS

**
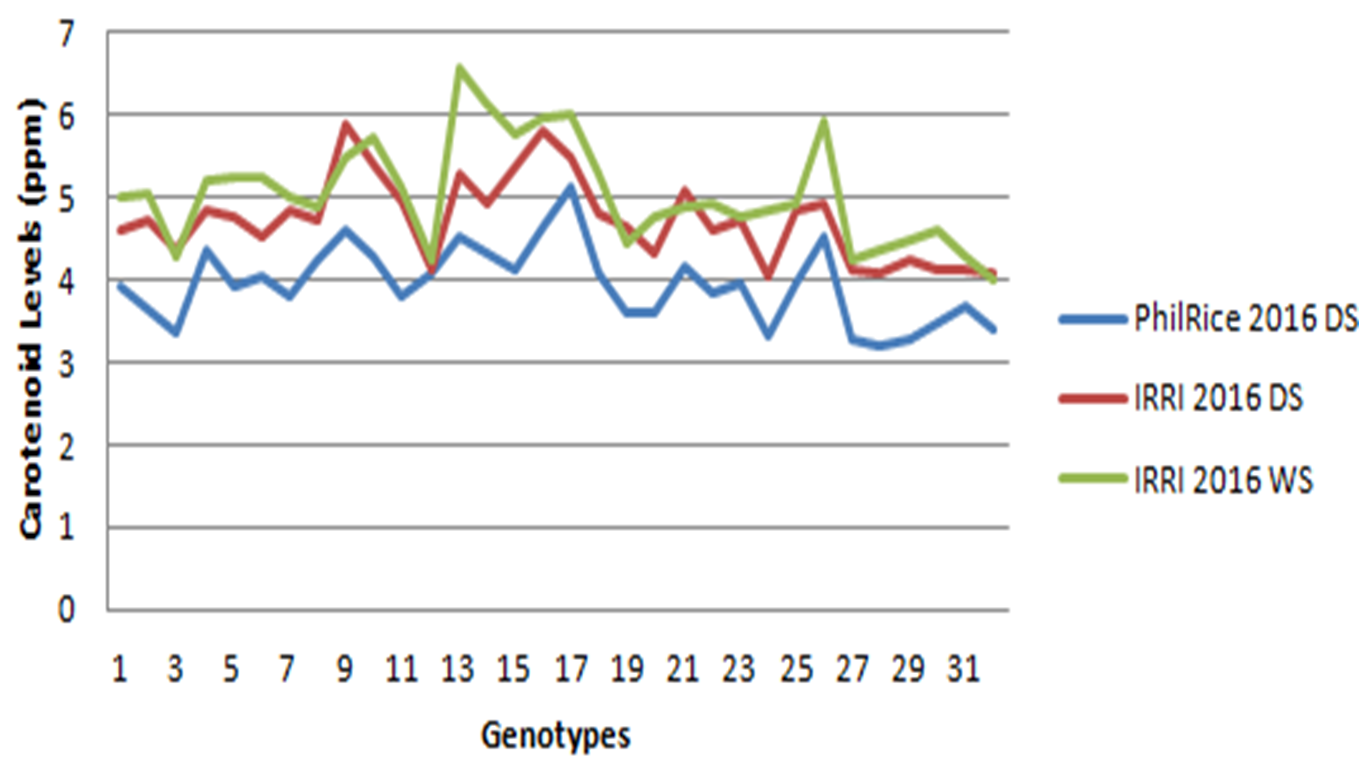
**

Fig S10. Carotenoid levels in GR2E PSBRc82 from different CTs

**
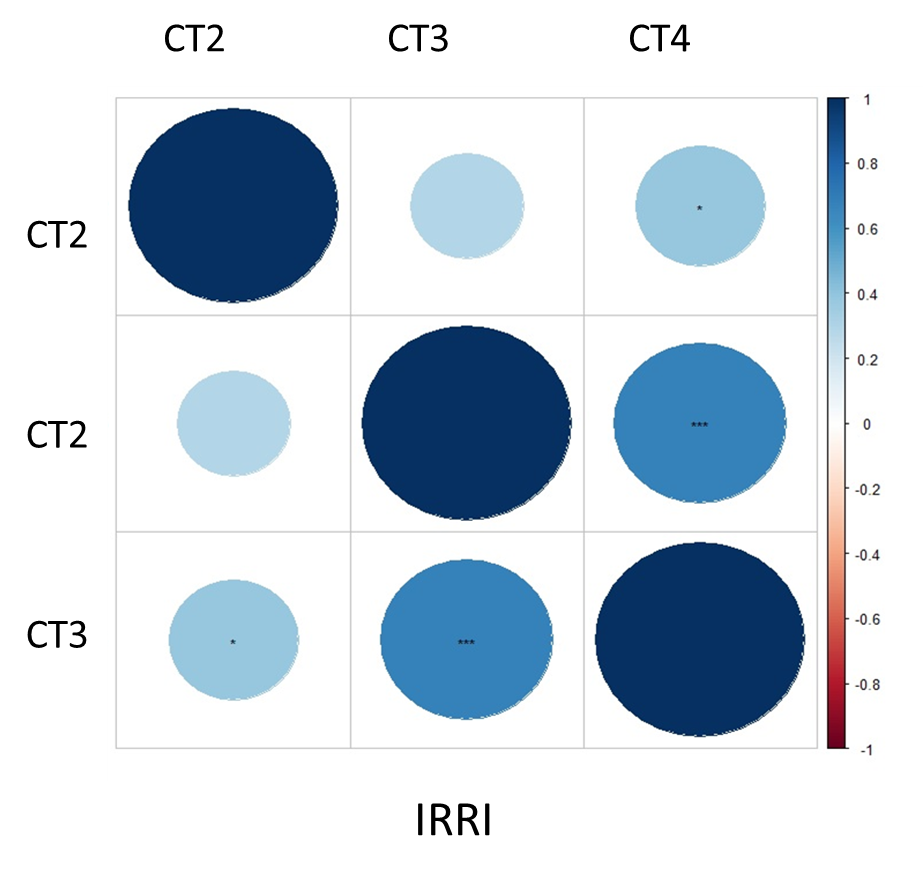
**

Fig S11. Correlation of carotenoids measured in different seasons in GR2E IR64

**
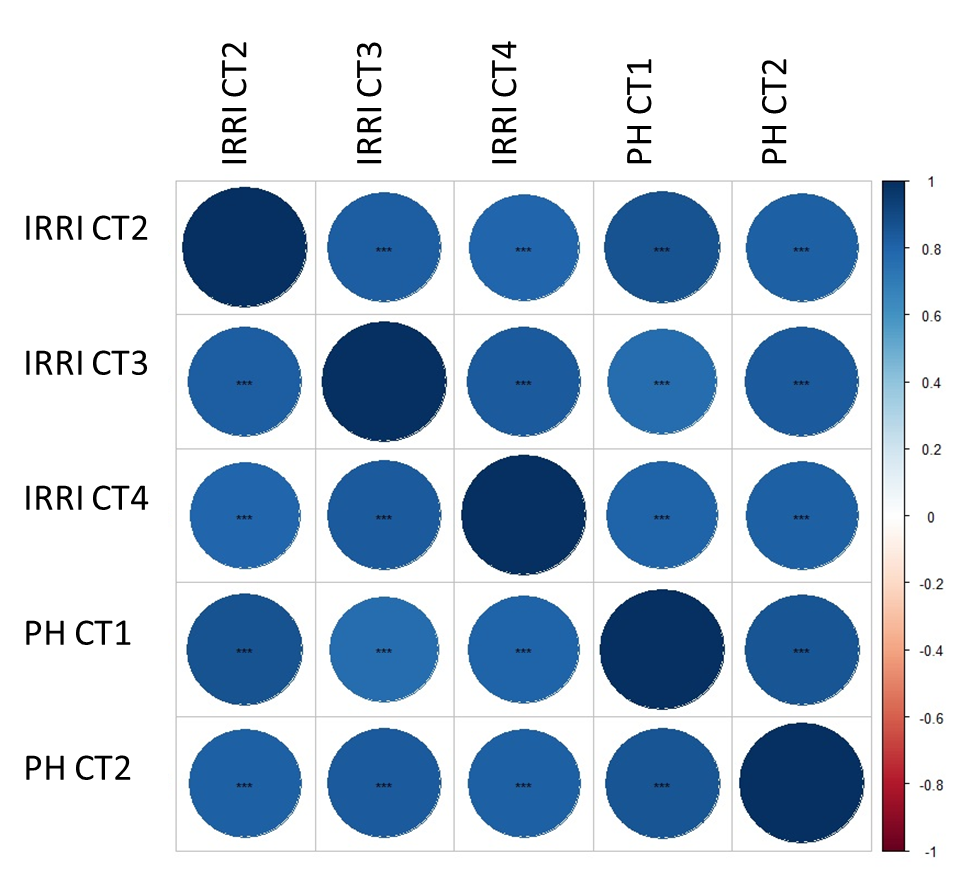
**

Fig S12. Correlation of carotenoids measured in different seasons in GR2E PSBRc82
